# Predicting Effects of Psychological Inflexibility/Experiential Avoidance and Stress Coping Strategies for Internet Addiction, Significant Depression, and Suicidality in College Students: A Prospective Study

**DOI:** 10.3390/ijerph15040788

**Published:** 2018-04-18

**Authors:** Wei-Po Chou, Cheng-Fang Yen, Tai-Ling Liu

**Affiliations:** 1 Department of Psychiatry, Kaohsiung Medical University Hospital, Kaohsiung 807, Taiwan; webber1007@gmail.com; 2 Department of Psychiatry, Tsyr-Huey Mental Hospital, Kaohsiung Jen-Ai’s Home, Kaohsiung 831, Taiwan; 3 Department of Psychiatry, School of Medicine, and Graduate Institute of Medicine, College of Medicine, Kaohsiung Medical University, Kaohsiung 807, Taiwan

**Keywords:** psychological inflexibility/experiential avoidance, stress coping strategies, internet addiction, depression, suicidality

## Abstract

The aims of this study were to evaluate the predicting effects of psychological inflexibility/experiential avoidance (PI/EA) and stress coping strategies for Internet addiction, significant depression and suicidality among college students during the follow-up period of one year. A total of 500 college students participated in this study. The level of PI/EA and stress coping strategies were evaluated initially. One year later, 324 participants were invited to complete the Chen Internet Addiction Scale, Beck Depression Inventory-II and the questionnaire for suicidality to evaluate depression symptoms and internet addiction and suicidality. The predicting effects of PI/EA and stress coping strategies were examined by using logistic regression analysis controlling for the effects of gender and age. The results indicated that PI/EA at the initial assessment increased the risk of Internet addiction (OR = 1.087, 95% CI: 1.042–1.135), significant depression (OR = 1.125, 95% CI: 1.081–1.170), and suicidality (OR = 1.099, 95% CI: 1.053–1.147) at the follow-up assessment. Less effective coping at the initial assessment also increased the risk of Internet addiction (OR = 1.074, 95% CI: 1.011–1.140), significant depression (OR = 1.091, 95% CI: 1.037–1.147), and suicidality (OR = 1.074, 95% CI: 1.014–1.138) at the follow-up assessment. Problem focused and emotion-focus coping at the initial assessment was not significantly associated with the risks of Internet addiction, significant depression, and suicidality at the follow-up assessment. College students who have high PI/EA or are accustomed to using less effective stress coping strategies should be the target of prevention programs for IA (internet addiction), depression, and suicidality.

## 1. Introduction

Internet addiction (IA), depression, and suicidality are major mental health issues among college students [1,2,3]. Approximately 8–13% of college students [1] and 1.4–20.8% of adolescents [4,5,6,7,8] have experienced IA during their lifetime. The highest prevalence was 20.8% in Taiwan (Yen et al., 2007) and the lowest prevalence was 1.4% in Finland [5]. People with IA experience various psychological distress symptoms [9], such as depression [10], suicidality [11], social anxiety [12], and low self-esteem [8,13,14]. Depression is common in college students and affects approximately 37% of college students in Taiwan [15]. Depression can cause functional impairment in multiple areas, such as school performance and behavior, peer relationships, and family relationships [16]. The costs of depression are substantial in terms of life and financial losses [15,16]. Suicide is the second leading cause of mortality in Taiwan among people aged 15–24 years [17]. Identifying factors that predict IA, depression, and suicidality can be helpful for developing prevention programs.

Both the psychological inflexibility/experiential avoidance (PI/EA) and strategies that individuals choose to cope with stress are the results of development since childhood and adolescence. The concept of PI/EA refers to the characteristic that is rigidly guided by psychological reactions rather than direct contingencies or personal values as well as unwillingness to experience unpleasant events or privations while pursuing one’s values and goals [18]. The definitions of psychological flexibility and psychological inflexibility are quite similar to those of experiential avoidance and acceptance [19]. People with high cognitive flexibility can rapidly and efficiently adapt to different situations [20,21], whereas people with high PI/EA were positively associated with multiple mental illnesses. For example, a cross-sectional study reported a positive association of PI/EA with IA [22]. Chawla reviewed previous experimental and correlational studies and reported a significant association of PI/EA with the development and maintenance of psychopathologies, including depression, negative emotion, and deliberate self-harm behavior [23]. However, no longitudinal study has examined the predictive value of PI/EA for IA, depression, and suicidality.

Dealing with stress is predominantly classified as a process, strategy, or style. The process approach involves subcategories called strategies or methods of coping with stress [24]. Problem-focused coping includes all the active efforts to manage stressful situations to modify or eliminate the sources of stress [25]. Emotion-focused coping includes all the regulative efforts to diminish the emotional consequences of stressful events [25]. Studies have found that maladaptive stress coping strategies had a cross-sectional association with IA [26,27]. Regarding depression, the coping strategies used by people with depression differ from those used by people without depression [24]. Studies have reported that less effective stress coping strategies had a positive association with an increased level of depression [28,29]. Maladaptive stress coping strategies were also significantly associated with the risk of suicidality [30]. A retrospective study demonstrated a negative association of problem-focused coping (both engagement and disengagement types) and a positive association of emotion-focused engagement coping with impulsive suicide attempt [31]. Developing effective coping behavior can reduce stress, help people solve personal problems, and maintain psychological balance and health [32]. A few longitudinal studies have discussed the predictive value of stress coping strategies for mental health issues. Previous longitudinal studies have used stress coping strategies to predict mortality and quality of life in hemodialysis patients [33], sexual risk behavior [34], and suicidal ideation [35]. The predictive values of Coping Orientation to Problems Experienced (COPE) for IA, depression, and suicidality should be determined.

This study investigated the predicting effects of PI/EA and stress coping strategies for IA, depression, and suicidality among college students during a 1-year follow-up period. We hypothesized that high PI/EA and less effective and emotion-focused stress coping predict a high risk of IA, depression, and suicidality 1 year later, whereas problem-focused stress coping predicts a low risk of IA, depression, and suicidality 1 year later.

## 2. Methods

### 2.1. Participants

Participants were recruited using an advertisement posted for college students aged between 20 and 30 years. Students willing to join the study could contact the research assistant through telephone, and the research assistant explained research procedures and screened volunteers’ eligibility. Eligible volunteers were called to our study room and informed regarding the research procedures again by the research assistant in person before they provided informed consent. Individuals who exhibited any deficits (e.g., intellectual disability or substance use) that prevented them from understanding the purpose of the study or from completing the questionnaires were excluded from the study. A total of 500 students (238 men and 262 women) from 67 colleges participated in this study. Their mean age was 22.1 years (standard deviation (SD): 1.8 years). Informed consent was obtained from all participants prior to assessment. This study was approved by the Institutional Review Board of Kaohsiung Medical University Hospital.

### 2.2. Measures

The Acceptance and Action Questionnaire-II (AAQ-II) [36] was revised from the original AAQ [37]. The AAQ-II consists of seven statements that represent various aspects of PI (e.g., “My painful experiences and memories make it difficult for me to live a life that I would value”) and EA (e.g., “I am afraid of my feelings”). The participants were asked to rate each of these statements on a scale of 1 (*never true*) to 7 (*always true*) based on their current experiences. A higher total score indicates a higher level of PI and EA. A study reported that the AAQ-II has adequate internal consistency and convergent and divergent validity [36]. The Cronbach’s α of the AAQ-II in the present study was 0.88.

#### 2.2.1. Coping Orientation to Problems Experienced

The 52-item self-administered COPE (Coping Orientation to Problems Experienced) [38] is composed of 13 scales, of which five measure problem-focused coping (active coping, planning, suppression of competing activities, restraint coping, and seeking instrumental social support), five measure emotion-focused coping (seeking emotional social support, positive reinterpretation, acceptance, denial, and turning to religion), and three measure coping responses that are generally less effective than the aforementioned responses (focus on and venting of emotions, behavioral disengagement, and mental disengagement). The COPE measures how people respond when they confront difficult or stressful events in their lives but do not cope with a specific stressful event. Every item is rated on a 4-point Likert scale. Higher total scale scores indicate that participants are more likely to cope with stress by using those strategies. The COPE has high reliability and validity [38]. The internal reliability (Cronbach’s α) of the 13 scales of the COPE in the present study ranged from 0.73 to 0.92.

#### 2.2.2. Chen Internet Addiction Scale 

We used the self-administered Chen Internet Addiction Scale (CIAS) to evaluate participants’ IA severity in the month preceding the study. [39]. The CIAS contains 26 items that are rated on a 4-point Likert scale, with scale scores ranging from 26 to 104 [39]. A higher total score indicates a more severe level of IA. The internal reliability (Cronbach’s α) of the CIAS in the present study was 0.93. According to the diagnostic criteria of IA, the 67/68 cutoff point of the CIAS has the highest diagnostic accuracy and accepted sensitivity and specificity [40]. Accordingly, participants with CIAS scores of 68 or more were classified as the IA group.

#### 2.2.3. Beck Depression Inventory-II

The 21-item Beck Depression Inventory-II (BDI-II) is a self-administered instrument used to assess the severity of depressive symptoms in the preceding 2 weeks [41]. A higher total BDI-II score indicates more severe depression. The Cronbach’s α for the BDI-II in the present study was 0.88. A total BDI-II score of 14 or higher indicates clinically significant depression [41]. Accordingly, participants with a total BDI-II score of 14 or more were identified as having significant depression.

#### 2.2.4. Suicidality

To evaluate the occurrence of suicidal attempt and four forms of suicidal ideation in the preceding year, participants were invited to complete a questionnaire containing the following questions from the epidemiological version of the Kiddie Schedule for Affective Disorders and Schizophrenia (Kiddie-SADS-E) [42]: (1) “Has there ever been a period of 2 weeks or longer when you thought a lot about death, including thoughts of your own death, somebody else’s death, or death in general?” (2) “Has there ever been a period of 2 weeks or longer when you had a desire to die?” (3) “Have you ever thought of attempting suicide?” (4) “Have you had a suicidal plan?” and (5) “Have you ever attempted suicide?” Although the original questionnaire was developed for measuring suicidality among children and adolescents, these questions for suicidality are not restricted to any one specific age group. Moreover, it has been used to assess suicidality among young adult gay and bisexual men in Taiwan [43]. Each question elicited a “yes” (scoring 1) or “no” (scoring 0) answer. Participants who responded to any question with a “yes” answer were classified to have suicidality.

### 2.3. Procedure and Statistical Analysis

In the initial assessment, the participants came to the research office. The researcher assistant explained the purpose of the research to the participants and then provided the participants with AAQ-II and COPE. Each measure contains the directions for participants to read before they completed and helps the participants feel free to provide honest answers.

One year later, the participants were invited to receive the follow-up assessment by telephone. The research staff called each participant for three times and classified those who did not answer the phone to be disconnected. Those who agreed to receive the follow-up assessment came to the research office again to complete the CIAS, BDI-II, and the questionnaire for suicidality. Participants received $NT 500.00 at the end of the assessment. The predicting effects of PI/EA and stress coping strategies at the initial assessment and 1 year later for IA, significant depression, and suicidality were examined using logistic regression analysis after controlling for the effects of sex and age. All statistical analyses were performed using SPSS 18.0 statistical software (SPSS Inc., Chicago, IL, USA). The OR is used to measure effect size, describing the strength of association between two binary data values. An odds ratio of more than 1 means that there is a higher odds of mental problems during follow up happening with PI/EA or IA initially. Odds ratio (OR) and its 95% confidence interval (CI) were used to present the statistical significance.

### 2.4. Ethical Considerations

Regarding the research was related with suicide, there was some ethical consideration as follow. Before research, we gave adequate education and training for all staff about the evaluation of suicidal risk. During research, we gave adequate explanation about the purpose of the research and got informed consent for all participants. We also inform all participants about how to manage risk of suicidal problems and provide available psychiatry service. After finishing the research, the staff would explain the result and conclusion of questionnaire to the participant individually. During whole course of research, we would transfer the participants who were in risk of suicidal to appropriate psychiatry service for full mental health evaluation. This study was supported by a grant awarded by Kaohsiung Medical University Hospital (KMUH103-3M3).

## 3. Results

A total of 324 college students (65.8%, 169 women and 155 men) received the follow-up assessment 1 year later. Of 176 participants who did not receive the follow-up assessment, 96 (54.5%) were disconnected, 48 (27.3%) refused participating in the follow-up assessment, and 32 (18.2%) had motivation but were not able to participate in the follow-up assessment due to work or army service. No difference in sex was found between participants who received and did not receive the follow-up assessment (*p* = 0.884), whereas participants who received the follow-up assessment were older than those who did not receive the follow-up assessment (*p* = 0.047). No differences in the levels of PI/EA (*p* = 0.488), problem-focused coping (*p* = 0.054), emotion-focused coping (*p* = 0.821) and less effective coping (*p* = 0.272) were found between participants who received and did not receive the follow-up assessment.

The PI/EA levels on the AAQ-II and stress coping strategies on the COPE at the initial assessment as well as the proportion of the participants with IA, significant depression, and suicidality at the follow-up assessment among the 324 participants are listed in Table 1. Of the total participants, 15.4%, 27.5%, and 17.0% had IA, significant depression, and suicidality at the follow-up assessment, respectively.

The results of the logistic regression analysis that was performed to examine the predicting effects of PI/EA and stress coping strategies at the initial and follow-up assessment for IA, significant depression, and suicidality are listed in Table 2. The results indicated that high PI/EA at the initial assessment increased the risk of IA (OR = 1.087, 95% CI: 1.042–1.135), significant depression (OR = 1.125, 95% CI: 1.081–1.170), and suicidality (OR = 1.099, 95% CI: 1.053–1.147) at the follow-up assessment. The use of less effective stress coping strategies at the initial assessment also increased the risk of IA (OR = 1.074, 95% CI: 1.011–1.140), significant depression (OR = 1.091, 95% CI: 1.037–1.147), and suicidality (OR = 1.074, 95% CI: 1.014–1.138) at the follow-up assessment. The use of problem-focused and emotion-focused coping strategies at the initial assessment was not significantly associated with the risk of IA, significant depression, and suicidality at the follow-up assessment.

## 4. Discussion

Identifying the predictors of mental health issues is one of the first steps to develop prevention programs. To the best of our knowledge, the present study is one of the first prospective study to examine the predictive value of stress coping strategies for IA and depression. This is also the first study to examine the predictive value of PI/EA for IA, depression, and suicidality. The results of this study revealed that high PI/EA and use of less effective stress coping strategies at the initial assessment increased the risk of IA, significant depression, and suicidality 1 year later.

A significant association of PI/EA with depression, anxiety, and substance use was reported in college students [44]. A study also demonstrated an association of PI/EA with deliberate self-harm behavior [23,44] and suicidality [45]. Chou and colleagues reported a positive association of PI/EA with IA [22]. Regarding the biological aspect, a study hypothesized that dopamine plays a crucial role in the development and maintenance of IA [46]. Cognitive flexibility has been associated with dopamine in many ways, such as through the dopamine receptor [47], dopaminergic signal function and control [48], and dopamine transporter genotype. Dopaminergic activity may play common roles in cognitive flexibility and IA. PI/EA is one of the important indicators of cognitive flexibility [19,49,50,51,52].

Interpersonal difficulty can be another factor that can explain the predictive value of PI/EA for IA, depression, and suicidality. PI/EA was positively associated with interpersonal relationship issues [51], which may increase the risk of depressive mood [53] and further increase the risk of suicidality [2,3]. The Internet provides a safe virtual environment for the unmet need of social requirement because the online space provides a rewarding sense of belonging, warmth, and well-being. These characteristics of the Internet may attract young adults with high PI/EA to overuse the Internet and therefore increase their risk of IA.

Addictive behaviors and stress are linked to each other in multiple aspects. Carey found that several stress-related hormones, such as cortisol, dopamine, and serotonin, may account for the association between addictive behaviors and maladaptive stress coping strategies [54,55,56,57]. Addictive behaviors are often initiated as a maladaptive mechanism for coping with stress [54]. The results of the present study confirmed that less effective stress coping strategy predicted the risk of IA 1 year later. Less effective stress coping strategy included focusing on and venting emotions, behavioral disengagement, and mental disengagement. People who are in the habit of using less effective stress coping strategy may overuse the Internet in an attempt to disengage from or vent about stressful events. The Internet can also serve as an inexpensive method to achieve immediate reinforcement. Internet overuse not only makes users preoccupied with the satisfaction obtained from the virtual world and increases the risk of IA but also increases the difficulties encountered in the real world.

The results of this study confirmed that less effective stress coping strategy predicted a high risk of significant depression and suicidality 1 year later. Many cross-sectional studies have reported a positive association of less effective stress coping strategy with depression [28,29,30]. Less effective stress coping strategy may result in further difficulties in the real world and thus deteriorate young adults’ emotional states. The risk of suicidality may also increase in the vicious circle of persistent stress, ineffective coping, and negative emotion.

Our study has some limitations that should be addressed. First, the data were exclusively self-reported, and we did not obtain additional information regarding mental diagnosis or treatment history. Second, we did not measure the levels of IA, depression, and suicidal tendency at baseline and thus could not determine the effect of PI/EA and stress coping strategies on changes in IA, depression, and suicidal tendency in the 1-year period. The conclusion of causality was also prohibited. Third, although the participants recruited from community are more representative compared with those recruited from clinical units, the volunteers may have various motivations for participating in the study. Moreover, their various backgrounds may lead to extraneous variables that we cannot control. Fourth, the depression and suicide and internet addiction were correlated with addictive disorder. This research did not include addictive disorder for evaluation and analysis. Fifth, the participants were informed about available service for their possible problems because the research team emphasis on ethical consideration. There was possibility that the participants may be more aware of their own problems and over report their problems.

The findings of the current study show that high PI/EA and use of less effective stress coping strategies increased the risk of IA, significant depression, and suicidality 1 year later in the college students. Mental health and education policy-makers may consider evaluating stress coping strategy and PI/EA of college student as predictive value of IA, depression and suicidality. Thus there may be benefit to provide those students with ineffective stress coping and psychological inflexibility with more counselling services and support from mental health professionals. The acceptance and commitment therapy uses a variety of strategies with behavioral changes and commitment to cultivate psychological flexibility [50] tress in school is usually high in Chinese society. To design stress reduction workshops that build on the existing effective stress coping strategies of college students could be helpful to manage stress [58].

## 5. Conclusions

In summary, the results of the present study indicated high PI/EA and use of less effective stress coping strategies increased the risk of IA, significant depression, and suicidality 1 year later in the college students. College students who have high PI/EA or are accustomed to using less effective stress coping strategies should be the target of prevention programs for IA, depression, and suicidality. Mental health and education professionals should motivate college students to cope with stress by using effective strategy; mental health and education professionals should train students to increase their psychological flexibility and reduce their avoidant tendencies.

## Figures and Tables

**Table 1:** Demographic characteristics, predictors at the initial interview, and outcome variables.

Participant’s Characteristics	*n* (%)	Mean (SD)	Range
Gender			
Female	169 (52.2)		
Male	155 (47.8)		
Age (years)		22.3 (1.9)	20–29
Predictors			
Psychological inflexibility/experiential avoidance on the AAQ-II		20.2 (7.4)	7–46
Stress coping strategies on the COPE			
Problem-focused coping		60.7 (8.9)	39–80
Emotion-focused coping		55.6 (8.7)	35–79
Less effective coping		20.5 (5.1)	12–35
Outcome Variables			
Internet addiction	50 (15.4)		
Significant depression	89 (27.5)		
Suicidality	55 (17.0)		

Note: AAQ-II: Acceptance and Action Questionnaire-II; COPE: Coping Orientation to Problems Experienced

**Table 2:** Predicting effects of psychological inflexibility/experiential avoidance and stress coping strategies for Internet addiction, significant depression, and suicidality.

Predicting Effects	Internet Addiction				Significant Depression				Suicidality			
	OR	95% CI of OR	OR	95% CI of OR	OR	95% CI of OR	OR	95% CI of OR	OR	95% CI of OR	OR	95% CI of OR
Sex	1.162	0.622–2.170	1.119	0.603–2.076	0.669	0.392–1.141	0.641	0.382–1.075	1.029	0.558–1.898	0.967	0.530–1.764
Age	1.131	0.973–1.314	1.115	0.958–1.298	0.956	0.829–1.102	0.936	0.812–1.079	0.869	0.724–1.044	0.848	0.703–1.022
Psychological inflexibility /experiential avoidance	1.087	1.042–1.135			1.125	1.081–1.170			1.099	1.053–1.147		
Problem-focused coping			0.979	0.942–1.018			0.981	0.950–1.014			0.991	0.954–1.029
Emotion-focused coping			1.007	0.968–1.047			0.981	0.949–1.013			0.982	0.945–1.019
Less effective coping			1.074	1.011–1.140			1.091	1.037–1.147			1.074	1.014–1.138

Note: Number in red means 95% CI > 1.
